# Supplementary material for: Structural mechanisms of autoinhibition and substrate recognition by the ubiquitin ligase HACE1
Source: Nat Struct Mol Biol. 2024 Feb 8;31(2):364–77. doi: 10.1038/s41594-023-01203-4 (PMC10873202; doi:10.1038/s41594-023-01203-4)
Supplement: Supplementary file 1 — Supplementary Methods, Figs. 1–11 and References. [file 41594_2023_1203_MOESM1_ESM.pdf]

nature structural & molecular biology

Article https://doi.org/10.1038/s41594-023-01203-4

Structural mechanisms of autoinhibition and substrate recognition by the ubiquitin ligase HACE1

In the format provided by the authors and unedited

SUPPLEMENTARY METHODS

HACE1 expression in Sf9 cells

pFastBac-HACE1 was used for transposition into *E. coli* DH10EMBacY (Geneva Biotech). Insertion of *HACE1* into the bacmid DNA was verified by PCR and the purified bacmid DNA transfected into Sf9 insect cells using XtremeGENE HP (Sigma-Aldrich). After 3 days at 27°C, YFP-positive cells were observed by fluorescence microscopy and the V_0 baculoviruses harvested. For virus amplification, 25 mL of Sf9 cells at a density of 1.0 x 10^6 cells/mL were infected with 3 mL of the V_0 stock. The Sf9 suspension cultures were incubated for 3 days at 27°C, after which the V_1 stocks were harvested. For large-scale expression, 4 x 1 L of Sf21 suspension cultures in Sf-900 III SFM medium (Thermo Fischer Scientific) at a density of 1.0 x 10^6 cells/mL were infected with 2.5 mL of the V_1 stock (1:400). The infected Sf21 cultures were harvested after 3 days at 27°C and the cell pellets flash-frozen and stored at -80°C.

HACE1 expression in mammalian cells

HEK293F cells, grown in FreeStyle 293F expression medium (Thermo Fischer Scientific), were passaged to fresh medium on the day before transfection at a density of 2 x10^6 cells/mL and transferred to a TPP TubeSpin bioreactor 600. The next day, the appropriate volume of culture was centrifuged (5 min, room temperature, 100 × *g*) and the cell pellet resuspended in 40 mL of fresh FreeStyle 293F medium to a final density of 20 × 10^6 cells/mL. The purified pcDNA3.1-HACE1 plasmid DNA was added to a final concentration of 1.5 μg/million cells and mixed. Immediately, 3 μg/million cells of a 1 mg/mL PEI MAX stock solution (MW 40000; Polysciences) were added, the cultures swirled again and transferred to an incubator-shaker (37 °C, 8% CO_2, 180 rpm) (Minitron; INFORS HT) for 1 h. At the end of the transfection phase, the cultures were divided into 2 TPP TubeSpin bioreactors 600 and 380 mL of pre-warmed FreeStyle 293 medium was added to each bioreactor to obtain a final density of 1 x 10^6 cells/mL. The transfected HEK293F cultures were incubated for 72 h (37 °C, 8% CO_2, 70% humidity, 180 rpm), harvested, and the cell pellets flash-frozen and stored at -80°C.

HDX MS

Following incubation of the reactions at 25 °C, HDX was quenched by mixing 55 μL of the reaction with 55 μL of 400 mM KH_2PO_4/H_3PO_4, 2 M guanidine-HCl (pH 2.2) at 1 °C, before injection into an ACQUITY UPLC M-Class with HDX Technology [1] through a 50-μL loop. Non-deuterated samples were prepared with an H_2O-based buffer. Samples were guided with water + 0.1% (v/v) formic acid ('eluent A'); 100 μL/min) to a porcine pepsin column at 12 °C and peptides collected on a trap column (2 mm x 2 cm; 0.5 °C) of POROS 20 R2 (Thermo Fisher Scientific). After 3 min, the trap was coupled to an ACQUITY UPLC BEH C18 1.7 μm 1.0 x 100 mm column (Waters) at 0.5 °C and peptides eluted

with an acetonitrile + 0.1% (v/v) formic acid gradient at 30 μL/min (0-7 min/95-65% A, 7-8 min/65-15% A, 8-10 min/15% A). MS was performed with a Synapt G2-Si mass spectrometer (Waters) using electrospray ionization (capillary temperature 250 °C; spray voltage 3.0 kV) and spectra acquired with MassLynX MS 4.1 (Waters) over 50-2000 *m/z* in enhanced high-definition MS ($HDMS^E$) [2] and high-definition MS mode for non-deuterated and deuterated samples, respectively. For lock-mass correction [Glu1]-fibrinopeptide B standard (Waters) was used. During separation, the pepsin column was washed 3 times with 80 μL of 0.5 M guanidine-HCl in 4% (v/v) acetonitrile. Blank runs were performed between each sample and measurements in triplicates.

Peptides were identified with ProteinLynx Global SERVER 3.0.1 and DynamX 3.0 (both Waters) from the non-deuterated samples acquired with $HDMS^E$, using low-energy, elevated-energy and intensity thresholds of 300, 100 and 1000 counts, respectively, and matched with a database containing the RAC1 Q61L, HACE1, porcine pepsin, and their reversed sequences (peptide tolerance = automatic; fragment tolerance = automatic; min. fragment ion matches per peptide = 1; min. fragment ion matches per protein = 7; min, peptide matches per protein = 3; max. hits to return = 20; max. protein mass = 250000; primary digest reagent = non-specific; missed cleavages = 0; false discovery rate = 100). For HDX quantification (DynamX), peptides had to be identified in ≥1/3 of the non-deuterated samples with ≥5000 counts; ≤40 residues length, ≥2 products and 0.05 products/residue, mass error ≤25 ppm, and retention time tolerance 0.5 min. Spectra were excluded, if required, e.g., for low signal-to-noise ratios or for overlapping peptides prohibiting assignment of isotopic clusters. Residue-specific deuterium uptake was calculated (DynamX). If a residue was covered by a single peptide, uptake was equal to that of the whole peptide. For overlapping peptides for a given residue, uptake was determined by the shortest peptide covering the residue. If multiple peptides had the shortest length, the peptide with the residue nearest the C-terminus was used.

SUPPLEMENTARY FIGURES (pages 3-13, below)
SUPPLEMENTARY REFERENCES (page 13, below)

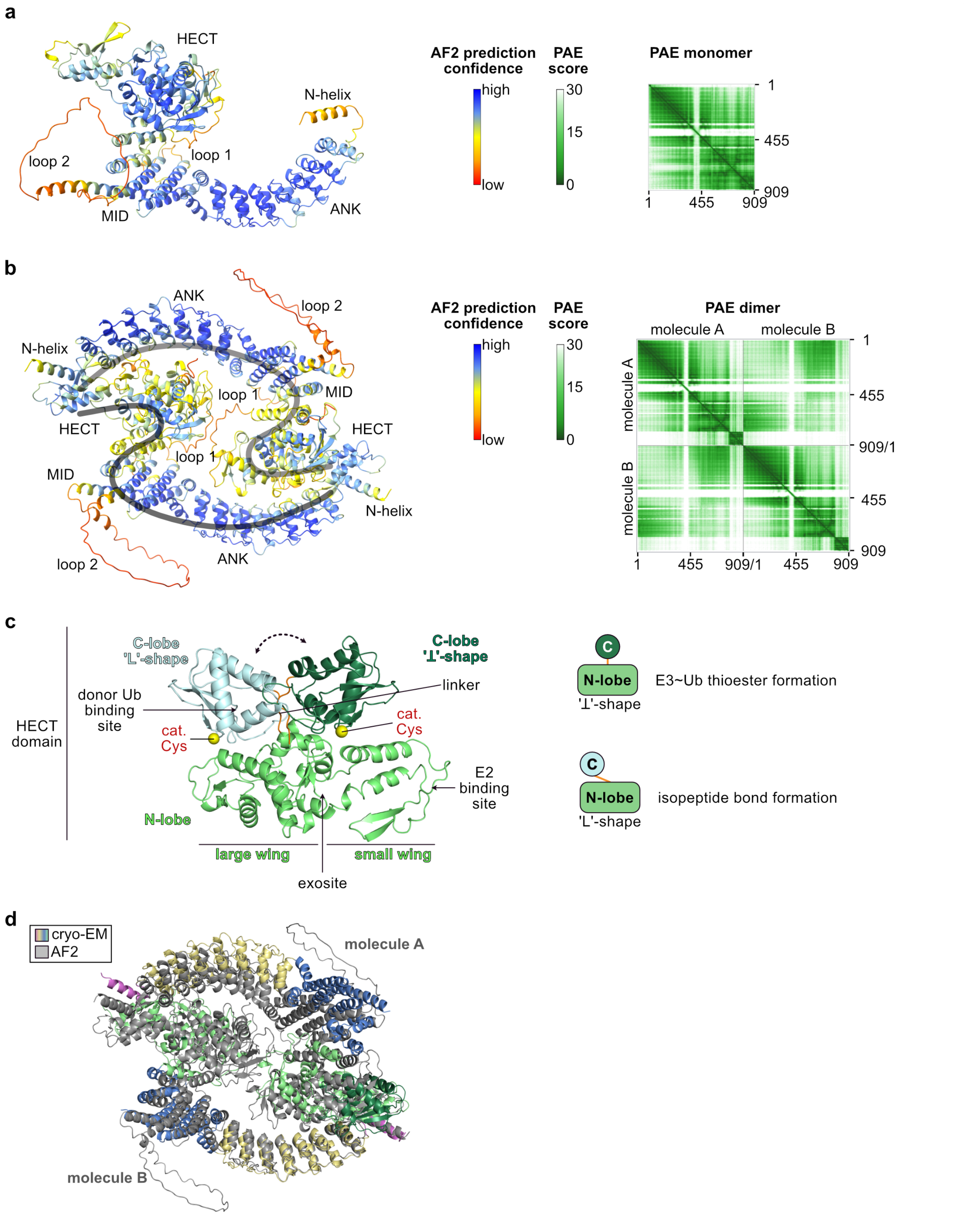

Supplementary Fig. 1. HECT domain architecture and structure predictions of HACE1
(a) Top-ranked, AF2 [3] structure prediction of HACE1 FL (pLDDT = 82.2), colored according to prediction confidence (left); predicted aligned error (PAE) scores (right). The conformations of the N-helix and loops are uncertain. Domains are labeled as in Fig. 1c.
(b) Top-ranked, AF2 structure prediction of a HACE1 FL dimer (weighted ipTM + pTM = 0.51), colored according to prediction confidence (left); predicted aligned error (PAE) scores (right).
(c) HECT domain of HACE1 in the inverted-T-conformation, extracted from the cryo-EM structure (molecule A; Fig. 1d); the C-lobe is additionally shown in the L-conformation, modelled based on a superposition with the HECT domain of HUWE1 (PDB ID: 6XZ1 [4]). Relevant regions and features are labeled (left). Cartoon representation of the two states of the HECT domain, as required for the specified catalytic steps (right)
(d) Superposition of the AF2-based model from (b) with the cryo-EM structure of HACE1 FL reported here (colored as in Fig. 1d); the ANKs of molecule B were superposed.

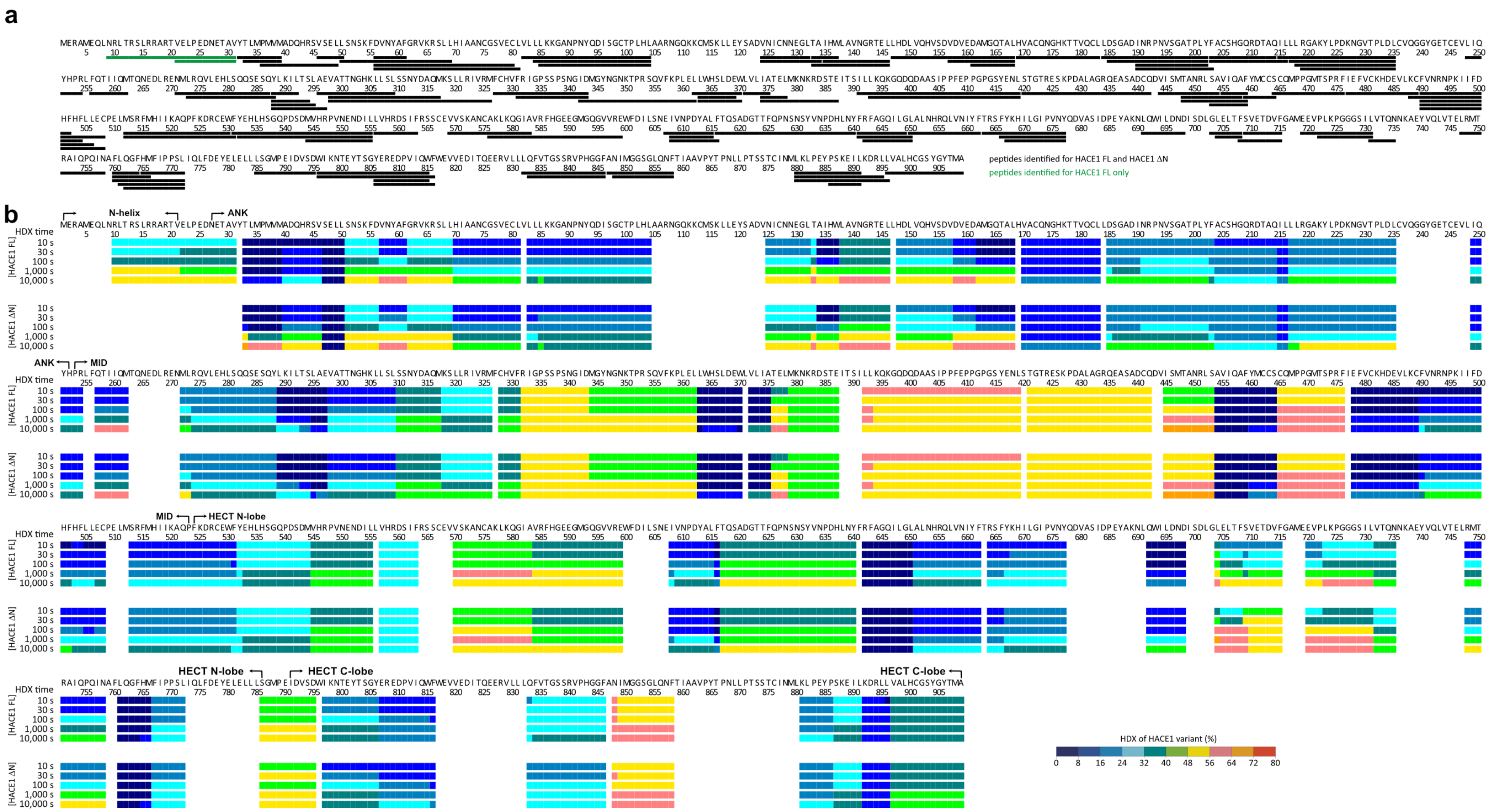

Supplementary Fig. 2. Individual HDX MS analyses of HACE1 FL and ΔN

(a) Amino acid sequence coverage upon proteolytic digestion of HACE1 FL and ΔN; each bar represents an identified peptide.

(b) Residue-specific HDX of HACE1 FL and ΔN, respectively, at the indicated time points, shown along the amino acid sequence; domain boundaries are indicated. For related raw data, see Supplementary Data 1.

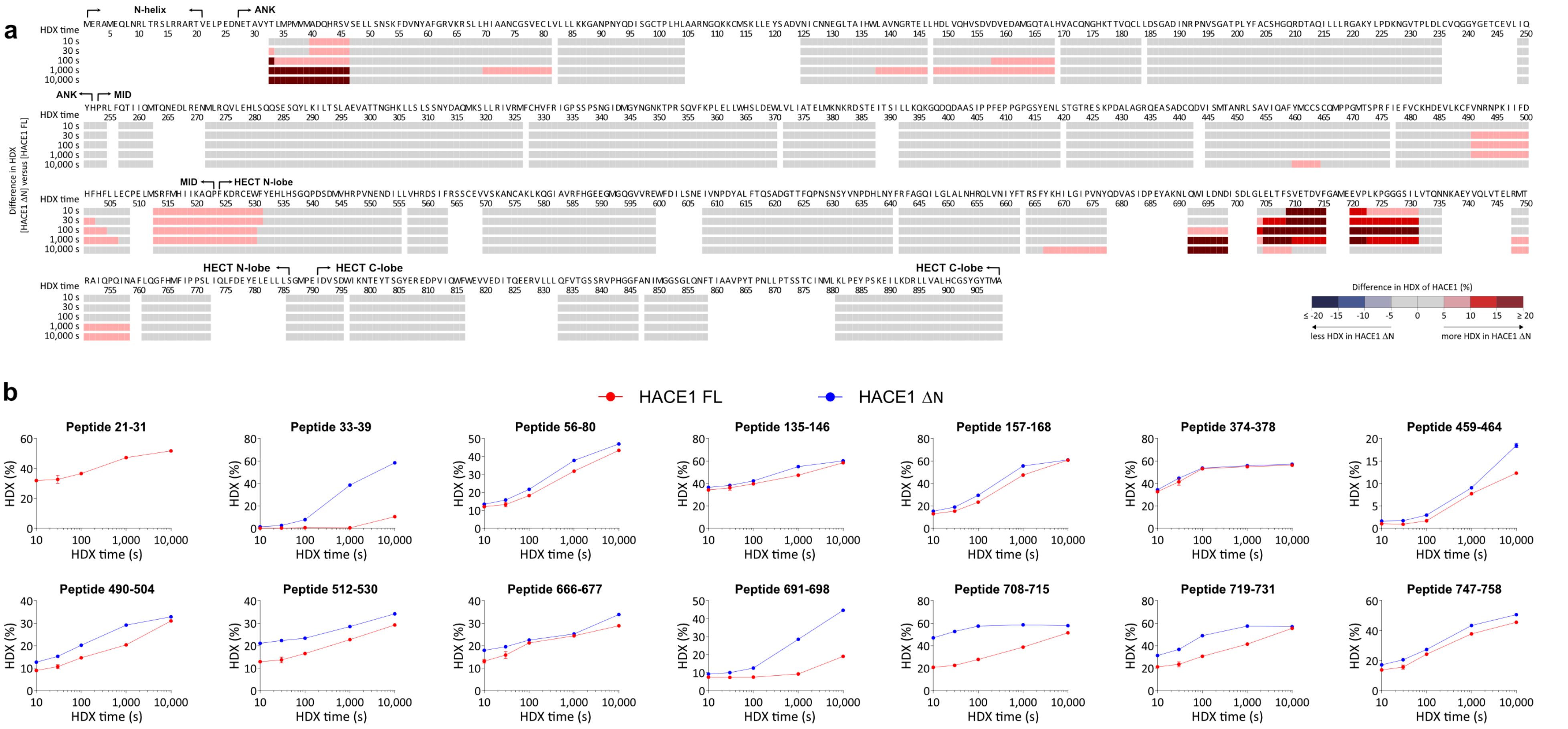

Supplementary Fig. 3. Differential HDX MS analyses, comparing HACE1 ΔN and FL

(a) HDX differences between HACE1 ΔN and FL at the indicated time points, shown along the amino acid sequence; domain boundaries are indicated. **(b)** Extent of HDX monitored over time for representative peptides derived from HACE1 FL and ΔN, respectively, plotted as the mean and standard deviations of 3 technical replicates. For related raw data, see Supplementary Data 1.

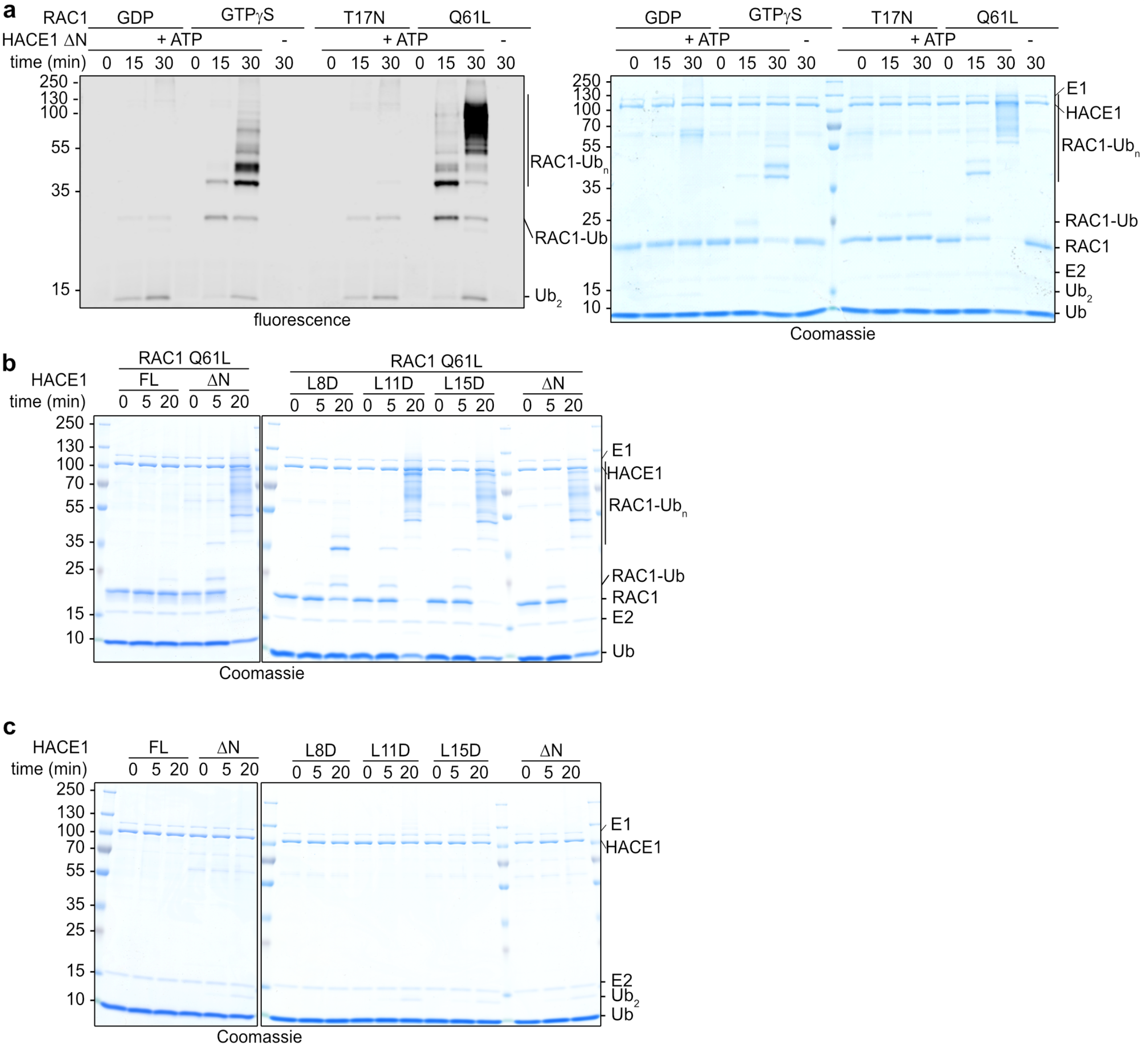

Supplementary Fig. 4. Functional analyses of HACE1 and RAC1 variants

(a) Reconstituted multi-turnover ubiquitination assay, monitoring the activity of HACE1 ΔN toward RAC1 variants and states; ubiquitinated products are visualized by fluorescence imaging (left), before Coomassie staining (right).

(b) Coomassie-stained image of the gel shown in Fig. 2c; 'FL' = WT

(c) Coomassie-stained image of the gel shown in Fig. 2d; 'FL' = WT

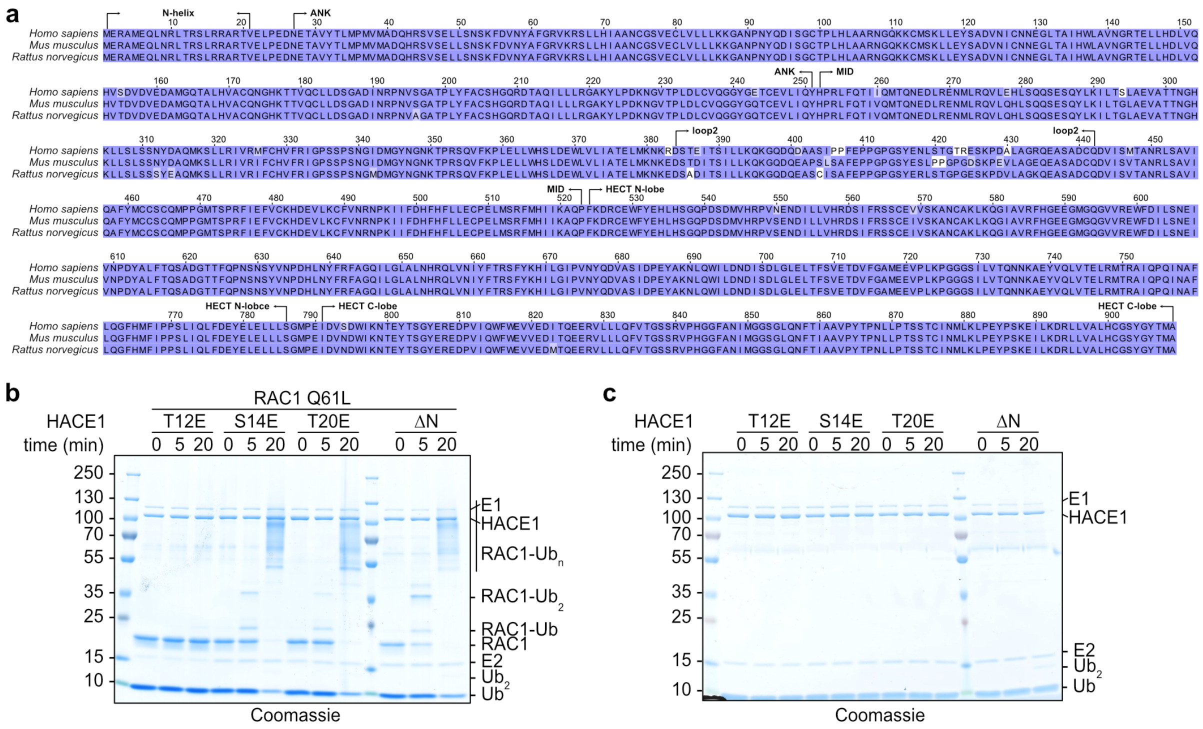

Supplementary Fig. 5. Amino acid sequence alignment and analyses of phospho-mimetic HACE1 variants

(a) Amino acid sequence alignment of HACE1 FL from human (NP_065822.2), mouse (NP_766061.2), and rat (NP_001102009.2); the sequence identity between human and mouse/rat is ~ 97%. The few amino acid changes accumulate in loop 2 (residues 384-442). The alignment was generated with PSI-BLAST [5], rendered with JalView [6] and colored according to the BLOSUM62 score [7].

(b) Coomassie-stained image of the gel shown in Fig. 3d.

(c) Coomassie-stained image of the gel shown in Fig. 3e.

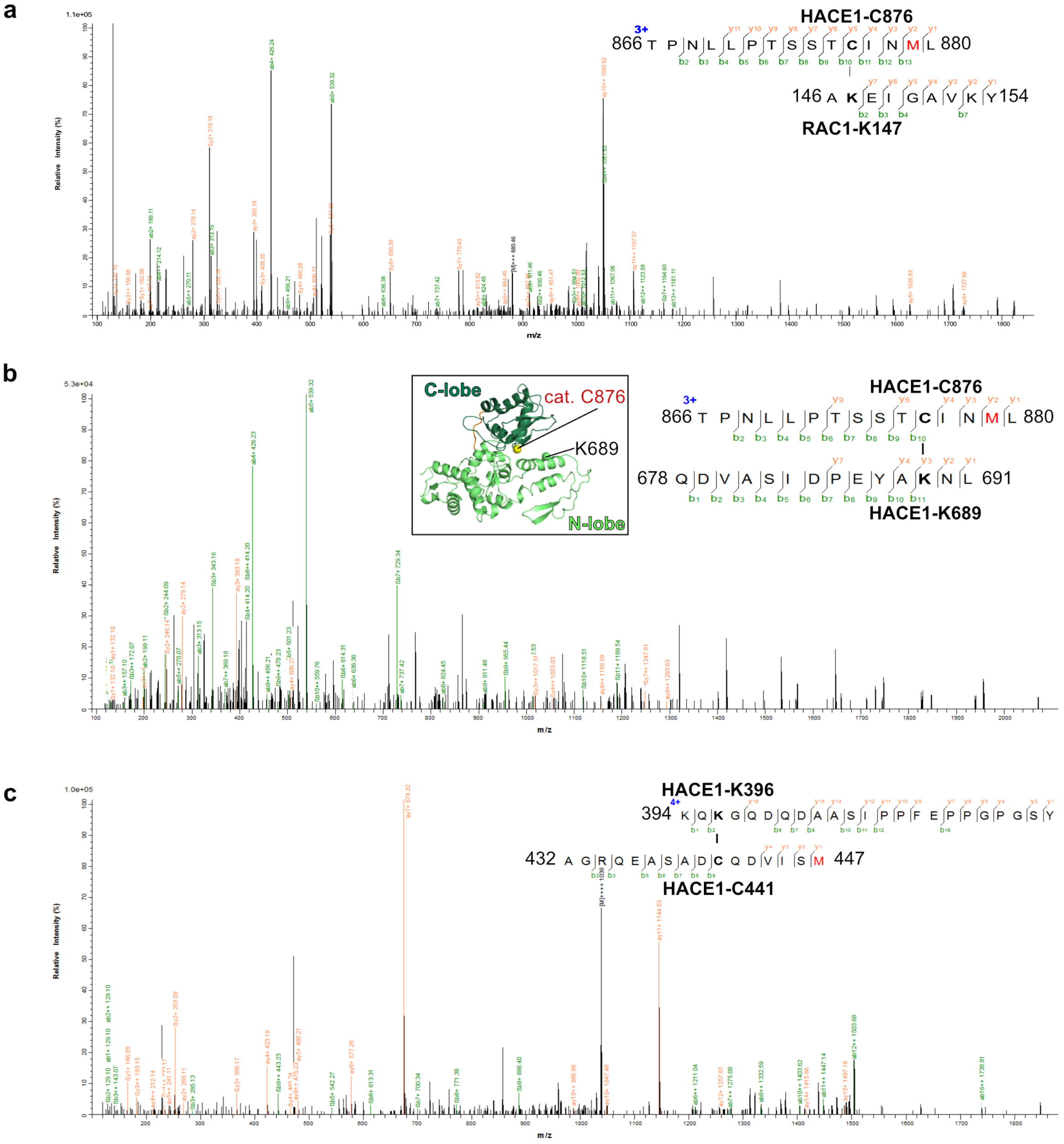

Supplementary Fig. 6. Mapping of SIA-crosslinks in HACE1 and RAC1 by MS

(a) Representative spectrum of the unique SIA-based HACE1-RAC1 crosslink (gel band 'XL-1'; Fig. 5b), involving the catalytic Cys 876 of HACE1 ΔN and the Lys 147-ubiquitination site of RAC1 Q61L, annotated with pLink [8]

(b) Representative spectrum of the intramolecular HACE1 crosslink (gel band 'HACE1'; Fig. 5b), involving the catalytic Cys 876 and Lys 869 of HACE1, annotated with pLink. The insert shows the proximity of the two sites within the inverted-T conformation of the HECT domain (HECT domain structure extracted from our cryo-EM structure of HACE1 FL)

(c) Representative spectrum of a HACE1-HACE1 crosslink (gel band 'XL-2'; Fig. 5b), involving two residues in the flexible, structurally uncharacterized loop 2 (Lys 396 and Cys 441), annotated with pLiNK.

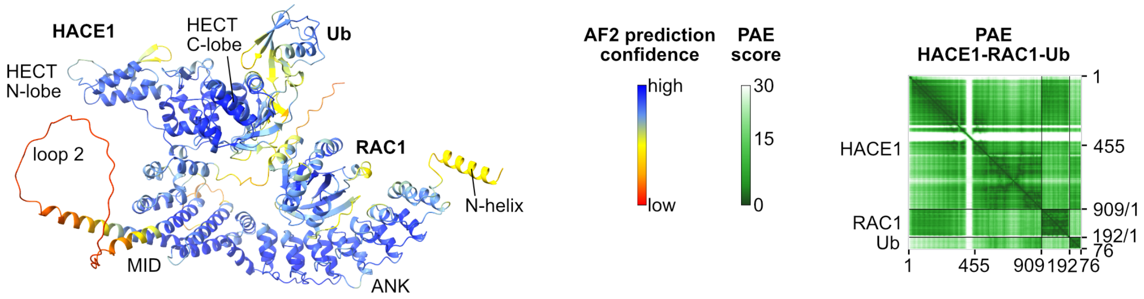

Supplementary Fig. 7. **Structure prediction of a ternary HACE1-RAC1-Ub complex**
AF2 structure prediction of a complex of HACE1, RAC1 and Ub (second best-ranked model; weighted ipTM + pTM = 0.72), colored according to prediction confidence (left); predicted aligned error (PAE) scores (right); note, that the top-ranked model did not have the HECT C-lobe in the L-conformation, nor did predictions of a binary HACE1-RAC1 complex.

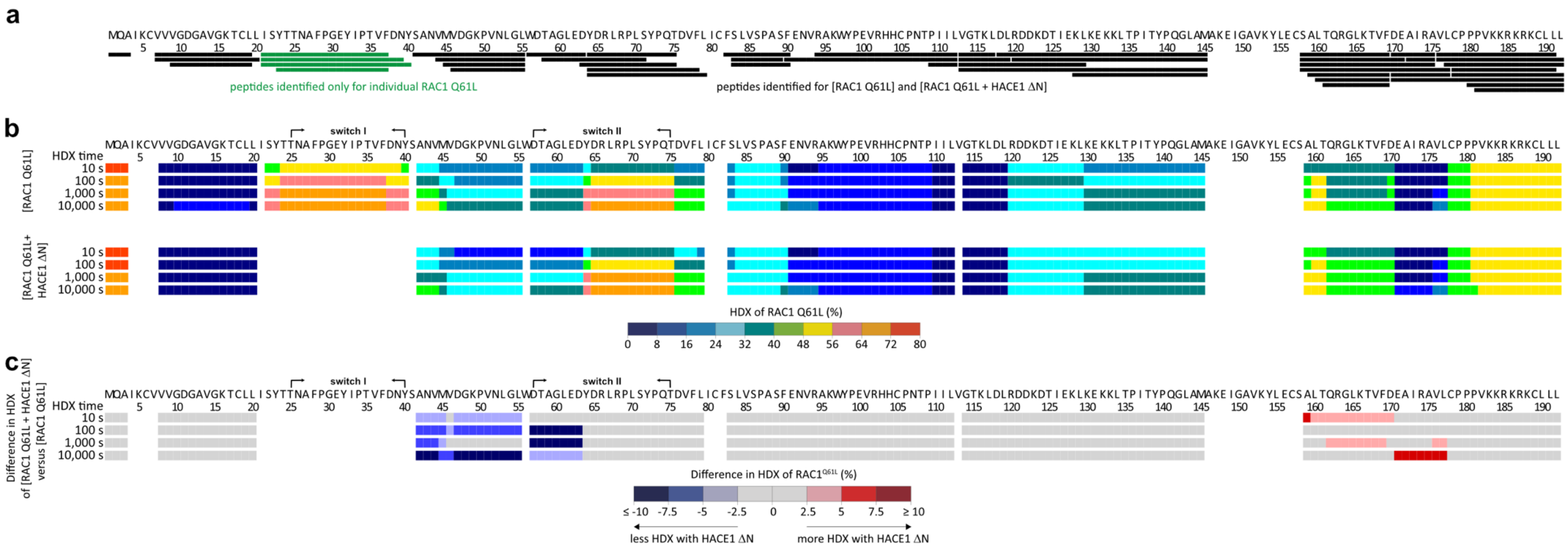

Supplementary Fig. 8. HDX MS analyses of RAC1 Q61L in the absence and presence of HACE1 ΔN
(a) Amino acid sequence coverage upon proteolytic digestion of RAC1 Q61L. Each bar represents an identified peptide.
(b) HDX of RAC1 Q61L in the absence and presence of HACE1 ΔN, respectively, at the indicated time points, shown along the amino acid sequence; the critical switch regions are marked. For raw data, see Supplementary Data 2.
(c) HDX differences between RAC1 Q61L in the absence and presence of HACE1 ΔN at the indicated time points, shown along the amino acid sequence; the critical switch regions are marked.

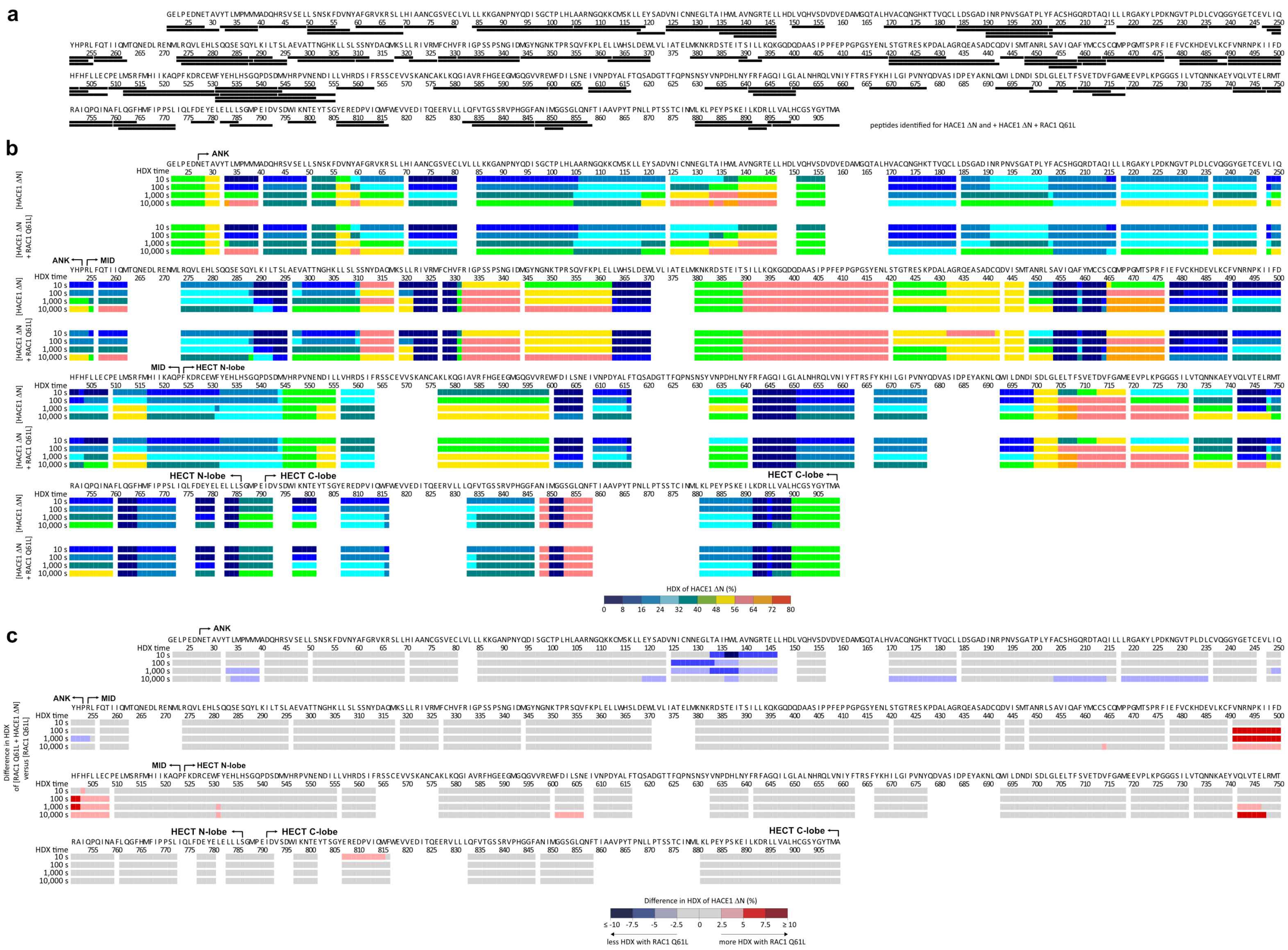

Supplementary Fig. 9. HDX MS analyses of HACE1 ∆N in the absence and presence of RAC1 Q61L, respectively

(a) Amino acid sequence coverage upon proteolytic digestion of HACE1 ∆N; each bar represents an identified peptide.

(b) HDX of HACE1 ∆N in the absence and presence of RAC1 Q61L at the indicated time points, shown along the amino acid sequence; domain boundaries are indicated. For related raw data, see Supplementary Data 2.

(c) HDX differences between HACE1 ∆N in the absence and presence of RAC1 Q61L at the indicated time points, shown along the amino acid sequence; domain boundaries are indicated.

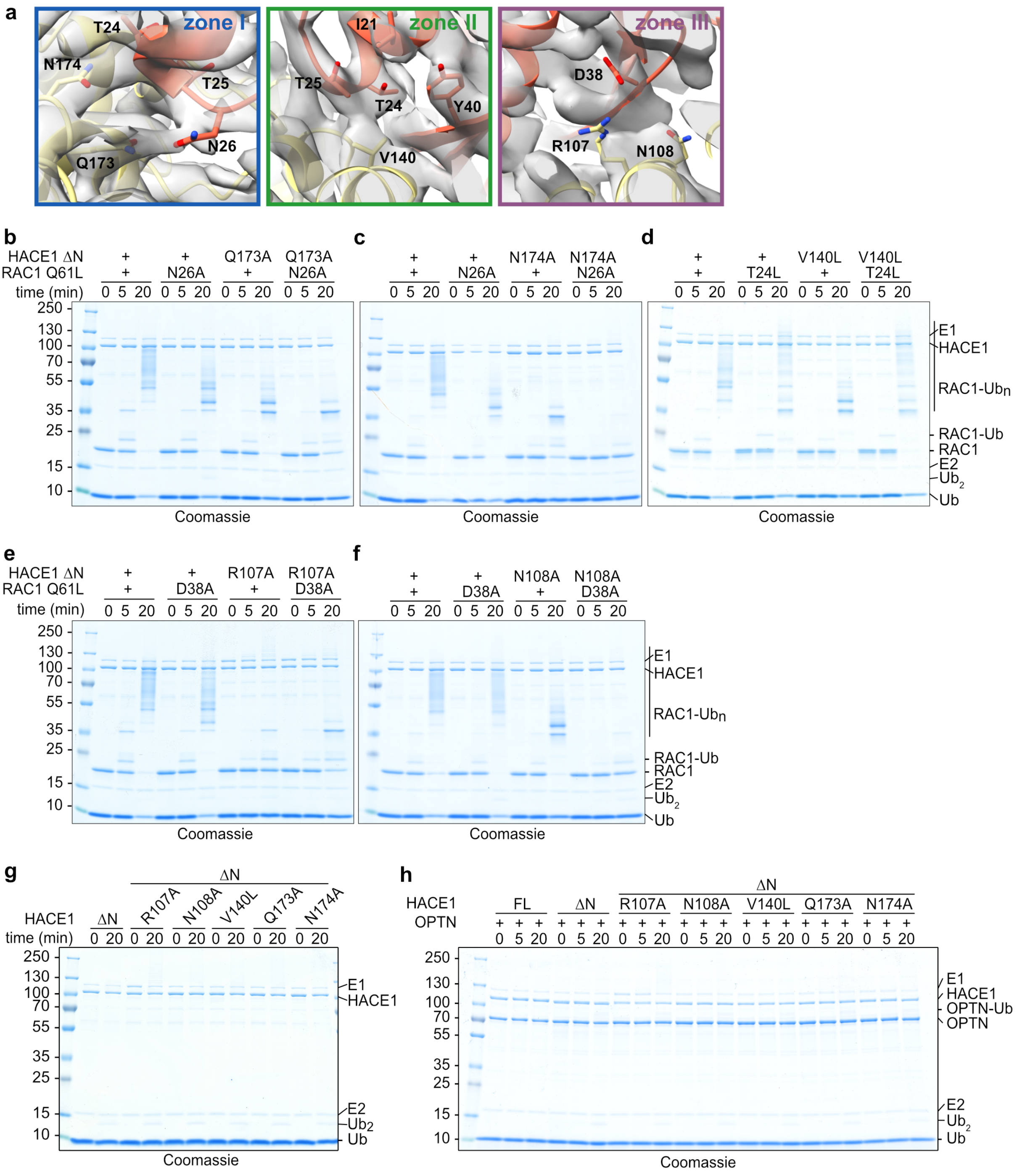

Supplementary Fig.10. Functional assays interrogating the HACE1-RAC1 interface

(a) Expanded views of the cryo-EM structure of the HACE1 ΔN-RAC1 Q61L complex (analogous to Fig. 6b), illustrating the definition of the analyzed amino acid side chains within the cryo-EM map (grey)

(b-h) Coomassie-stained images of the gels shown in Fig. 6c-h,j; 'FL' = W

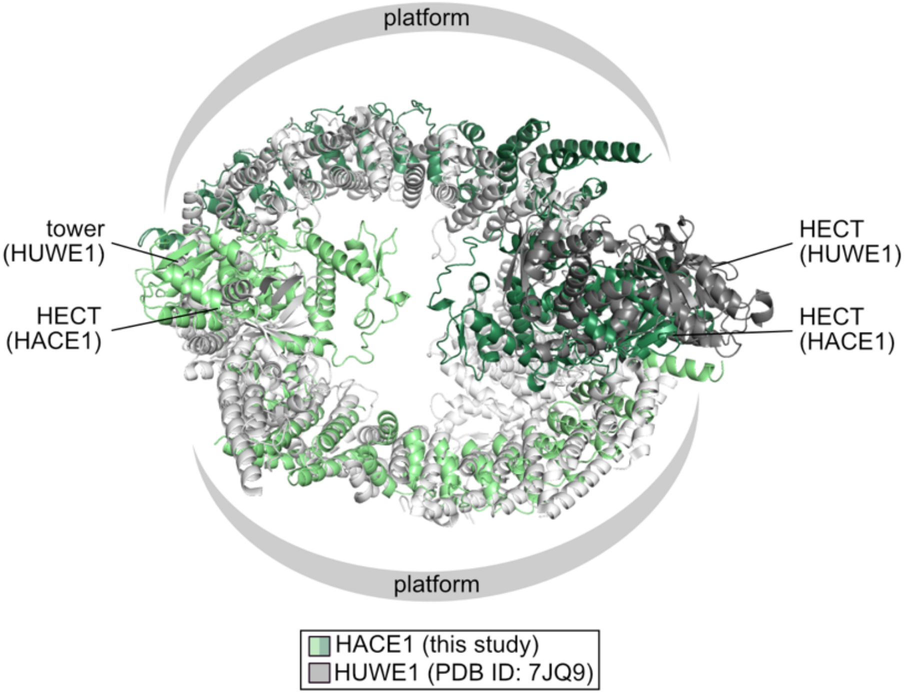

Supplementary Fig. 11. Global similarity of the HACE1 dimer to HUWE1
Manual superposition of the ring-like structures of HACE1 FL, determined here, and HUWE1 (PDB ID: 7JQ9 [9]), viewed from the top. The two HACE1 molecules are shown in different shades of green, HUWE1 in grey. Only selected domains are labeled for clarity.

SUPPLEMENTARY REFERENCES

1. Wales, T. E., Fadgen, K. E., Gerhardt, G. C. & Engen, J. R. High-Speed and High-Resolution UPLC Separation at Zero Degrees Celsius. *Anal. Chem.* **80**, 6815–6820 (2008).

2. Geromanos, S. J. *et al.* The detection, correlation, and comparison of peptide precursor and product ions from data independent LC-MS with data dependant LC-MS/MS. *Proteomics* **9**, 1683–1695 (2009).

3. Jumper, J. *et al.* Highly accurate protein structure prediction with AlphaFold. *Nature* **596**, 583–589 (2021).

4. Nair, R. M. *et al.* Reconstitution and Structural Analysis of a HECT Ligase-Ubiquitin Complex via an Activity-Based Probe. *ACS Chem. Biol.* **16**, 1615–1621 (2021).

5. Altschul, S. F. *et al.* Gapped BLAST and PSI-BLAST: a new generation of protein database search programs. *Nucleic Acids Res.* **25**, 3389–3402 (1997).

6. Waterhouse, A. M., Procter, J. B., Martin, D. M. A., Clamp, M. & Barton, G. J. Jalview Version 2-a multiple sequence alignment editor and analysis workbench. *Bioinformatics* **25**, 1189–1191 (2009).

7. Henikoff, S. & Henikoff, J. G. Amino-Acid Substitution Matrices From Protein Blocks. *Proc Natl Acad Sci USA* **89**, 10915–10919 (1992).

8. Chen, Z.-L. *et al.* A high-speed search engine pLink 2 with systematic evaluation for proteome-scale identification of cross-linked peptides. *Nat. Commun.* **10**, 3404–12 (2019).

9. Hunkeler, M. *et al.* Solenoid architecture of HUWE1 contributes to ligase activity and substrate recognition. *Mol. Cell* **81**, 3468-3480.e7 (2020).
