# Supplementary material for: Structural mechanisms of autoinhibition and substrate recognition by the ubiquitin ligase HACE1
Source: Nat Struct Mol Biol. 2024 Feb 8;31(2):364–77. doi: 10.1038/s41594-023-01203-4 (PMC10873202; doi:10.1038/s41594-023-01203-4)
Supplement: Supplementary file 2 — Reporting Summary [file 41594_2023_1203_MOESM2_ESM.pdf]

nature portfolio

Corresponding author(s): Sonja Lorenz

Last updated by author(s): Nov 23, 2023

Reporting Summary

Nature Portfolio wishes to improve the reproducibility of the work that we publish. This form provides structure for consistency and transparency in reporting. For further information on Nature Portfolio policies, see our Editorial Policies and the Editorial Policy Checklist.

Statistics

For all statistical analyses, confirm that the following items are present in the figure legend, table legend, main text, or Methods section.

n/a	Confirmed	
☐	☒	The exact sample size (n) for each experimental group/condition, given as a discrete number and unit of measurement
☐	☒	A statement on whether measurements were taken from distinct samples or whether the same sample was measured repeatedly
☒	☐	The statistical test(s) used AND whether they are one- or two-sided<br>*Only common tests should be described solely by name; describe more complex techniques in the Methods section.*
☒	☐	A description of all covariates tested
☒	☐	A description of any assumptions or corrections, such as tests of normality and adjustment for multiple comparisons
☐	☒	A full description of the statistical parameters including central tendency (e.g. means) or other basic estimates (e.g. regression coefficient) AND variation (e.g. standard deviation) or associated estimates of uncertainty (e.g. confidence intervals)
☒	☐	For null hypothesis testing, the test statistic (e.g. F, t, r) with confidence intervals, effect sizes, degrees of freedom and P value noted<br>*Give P values as exact values whenever suitable.*
☒	☐	For Bayesian analysis, information on the choice of priors and Markov chain Monte Carlo settings
☒	☐	For hierarchical and complex designs, identification of the appropriate level for tests and full reporting of outcomes
☒	☐	Estimates of effect sizes (e.g. Cohen's d, Pearson's r), indicating how they were calculated

Our web collection on statistics for biologists contains articles on many of the points above.

Software and code

Policy information about availability of computer code

Data collection	AcquireMP version 2023_R2.2 with IScat v1.58.0 and IScat Utils v 1.45.0 (Refeyn), SerialEM version 4.0 (University of Colorado Boulder), Image Reader LAS-1000 Pro version 2.6 (Fujifilm)
Data analysis	DiscoverMP version 2023_R2 with IScat v1.58.0 and IScat Utils v 1.45.0 (Refeyn), PhotoMol accessed 2023-09-05 (EMBL), Prism version 9.5.1 (733) (GraphPad), Warp version 1.0.9 (Dimitry Tegunov, Patrick Cramer), Relion version 3.1.0 (Sjors Scheres, MRC LMB), CryoSparc version 4.4.0+231114 (Structura Biotechnology), Pymol version 2.5.0 (Schrödinger), Phenix version 1.20.1-4487 (Lawrence Berkeley National Laboratory), ChimeraX version 1.6.1 (UCSF), AlphaFold monomer/multimer version 2.3.1 (DeepMind), ATSAS version 3.0.5 (EMBL), GASBOR version 2.3 (EMBL), SUPCOMB version 13+20 (EMBL), AllosMod-FoXS version main.1d1c5f6 (UCSF), Multi-FoXS version main.26c04d3 (UCSF), ImageStudio Lite version 5.2 (LI-COR)

For manuscripts utilizing custom algorithms or software that are central to the research but not yet described in published literature, software must be made available to editors and reviewers. We strongly encourage code deposition in a community repository (e.g. GitHub). See the Nature Portfolio guidelines for submitting code & software for further information.

Data

Policy information about availability of data

All manuscripts must include a data availability statement. This statement should provide the following information, where applicable:

- Accession codes, unique identifiers, or web links for publicly available datasets
- A description of any restrictions on data availability
- For clinical datasets or third party data, please ensure that the statement adheres to our policy

The cryo-EM structures of HACE1 FL and the HACE1 deltaN-RAC Q61L complex were deposited under PDB IDs 8PWL and 8Q0N; the maps under IDs EMD-17994 and EMD-18056, respectively. The HDX-MS data were deposited to the ProteomeXchange Consortium via the PRIDE partner repository, ID: PXD045837

Research involving human participants, their data, or biological material

Policy information about studies with human participants or human data. See also policy information about sex, gender (identity/presentation), and sexual orientation and race, ethnicity and racism.

Reporting on sex and gender	Not applicable.
Reporting on race, ethnicity, or other socially relevant groupings	Not applicable.
Population characteristics	Not applicable.
Recruitment	Not applicable.
Ethics oversight	Not applicable.

Note that full information on the approval of the study protocol must also be provided in the manuscript.

Field-specific reporting

Please select the one below that is the best fit for your research. If you are not sure, read the appropriate sections before making your selection.

☒ Life sciences ☐ Behavioural & social sciences ☐ Ecological, evolutionary & environmental sciences

For a reference copy of the document with all sections, see nature.com/documents/nr-reporting-summary-flat.pdf

Life sciences study design

All studies must disclose on these points even when the disclosure is negative.

Sample size	The data presented in the manuscript represent the averages and/or representatives of at least 3 independent replicates (see section "statistics and reproducibility: All gel-based analyses, including in-vitro and cell-based activity assays/IPs, of which representative results are displayed, were independently repeated at least 3 times with similar results.") . These sample sizes were chosen to generate data at sufficient depth and assess differences between conditions robustly. These sample sizes are sufficient, since the observed effects of interest are clearly detectable between conditions and robust across replicates. The error bars present in SAXS and HDX MS analyses are detailed in the text.
Data exclusions	No data were excluded.
Replication	All experiments were performed for at least n=3 independent samples, as described, and all attempts were successful. Immunoblots and enzyme assays were performed independently 3 times with similar results. Biochemical in-vitro experimets and functional cell-based assays were performed on separate and fully independent occassions and verified each other.
Randomization	Gel-based samples were run in different orders with the same result. Other than that, randomization is not relevant to this study, as no experimental groups were used.
Blinding	MS and structural data were analyzed with script-based pipelines, in which results are largely independent from interference of the researchers. The precise workflows are detailed in the methods section. Gel-based assays were replicated by different individuals. The investigators were not blinded, which is standard in this type of study due to multiple steps that require precise operations for accuracy and precision.

Reporting for specific materials, systems and methods

April 2023

We require information from authors about some types of materials, experimental systems and methods used in many studies. Here, indicate whether each material, system or method listed is relevant to your study. If you are not sure if a list item applies to your research, read the appropriate section before selecting a response.

Materials & experimental systems

n/a	Involved in the study
☐	☒ Antibodies
☐	☒ Eukaryotic cell lines
☒	☐ Palaeontology and archaeology
☒	☐ Animals and other organisms
☒	☐ Clinical data
☒	☐ Dual use research of concern
☒	☐ Plants

Methods

n/a	Involved in the study
☒	☐ ChIP-seq
☒	☐ Flow cytometry
☒	☐ MRI-based neuroimaging

Antibodies

Antibodies used

The following primary antibodies were used: anti-HA mouse monoclonal antibody (H9658; Sigma-Aldrich; dilution 1: 10,000); anti-GFP rabbit antiserum (132002; Synaptic Systems; 1: 1000); anti-HACE1 rabbit monoclonal antibody (EPR7962, ab133637; Abcam; 1: 500); and anti-Ub mouse monoclonal antibody P4D1 (sc-8017; Santa Cruz Biotechnology; 1: 1000). Fluorescently labeled donkey secondary antibodies included anti-mouse IRDye 680RD and anti-rabbit IRDye 800CW (926-68072 and 926-32213; LI-COR; 1: 20,000). For luminescence-based detection goat anti-rabbit HRP-linked antibody 7074 (Cell Signaling Technology; 1: 10000) was used.

Validation

Validation was performed by the manufacturer, as follows: ab133637 (abcam): WB; sc-8017 (Santa Cruz): IP, WB, IHC(P), ELISA, IF, FCM; 132 002 (Synaptic Systems): IP, WB, ICC, IHC, IHC(P), EM; H9658 (Sigma-Aldrich): IP, WB, ICC, ELISA; 926-32213 (LI-COR): WB; 926-68072 (LI-COR): WB; #7074 (Cell Signaling Technology): WB

Eukaryotic cell lines

Policy information about cell lines and Sex and Gender in Research

Cell line source(s)

Commercial HeLa Kyoto cells were a gift from Dr. Peter Lenart (MPI NAT, Goettingen, Germany); Sf9, HEK Flp-In, and HEK293F cells were purchased from Thermo Fischer Scientific.

Authentication

Cell lines were not authenticated beyond ensuring the presence of known antibiotic resistance markers within their genomes (by growth in the relevant antibiotics), monitoring the morphology and growth rates continuously, and PCR-amplifying the Flp-In locus from genomic DNA extracted from the cells (in case of HEK Flp-In).

Mycoplasma contamination

All cell lines were tested negative for mycoplasma contamination.

Commonly misidentified lines
(See ICLAC register)

No commonly misidentified lines were used.
