# Supplementary figures and images for: Structural mechanisms of autoinhibition and substrate recognition by the ubiquitin ligase HACE1
Source: Nat Struct Mol Biol. 2024 Feb 8;31(2):364–77. doi: 10.1038/s41594-023-01203-4 (PMC10873202; doi:10.1038/s41594-023-01203-4)

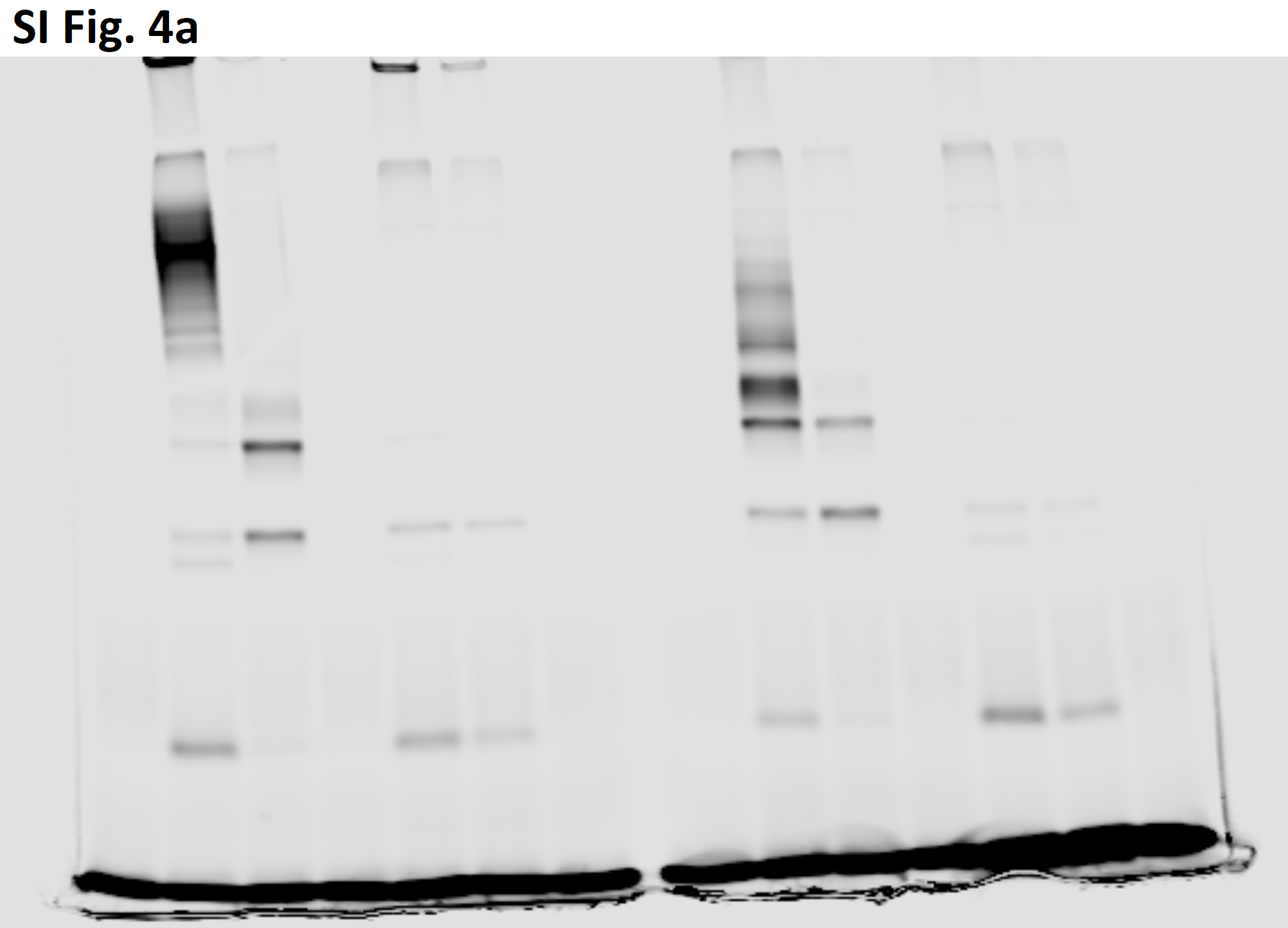

Supplement: Supplementary file 9 — Unprocessed gel. [file 41594_2023_1203_MOESM9_ESM.tif]

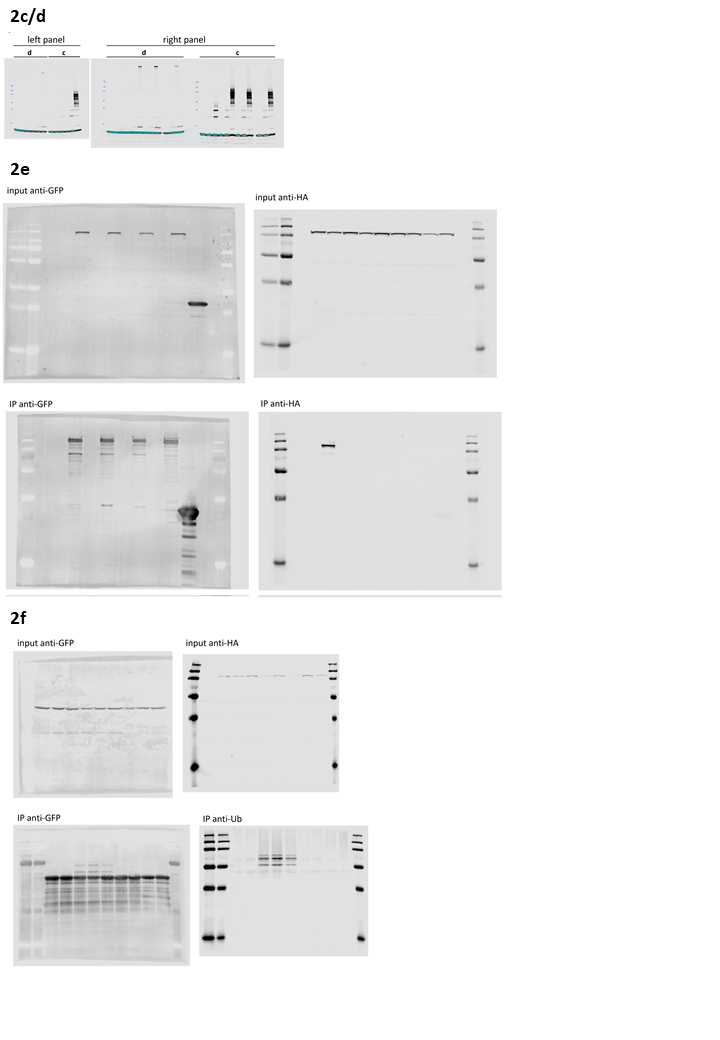

Supplement: Supplementary file 12 — Unprocessed Western blots. [file 41594_2023_1203_MOESM12_ESM.tif]

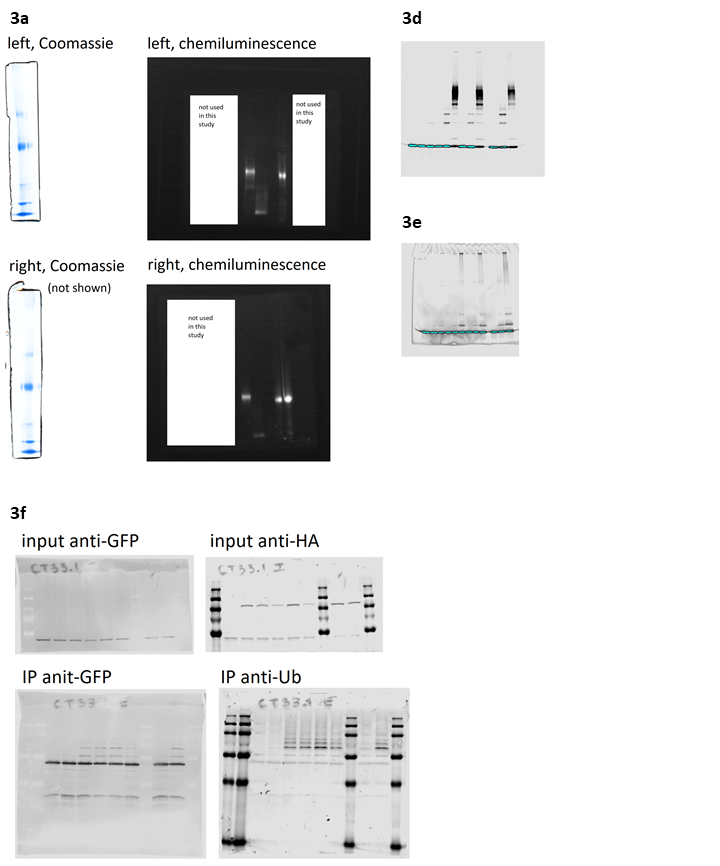

Supplement: Supplementary file 13 — Unprocessed gels and Western blots. [file 41594_2023_1203_MOESM13_ESM.tif]

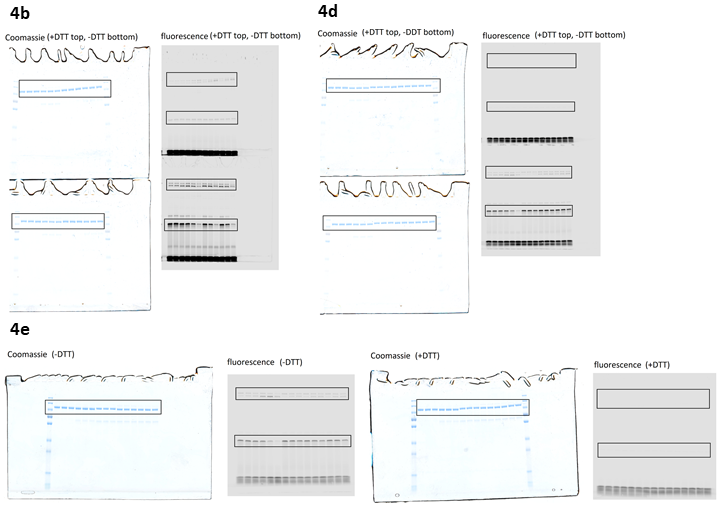

Supplement: Supplementary file 14 — Unprocessed gels. [file 41594_2023_1203_MOESM14_ESM.tif]

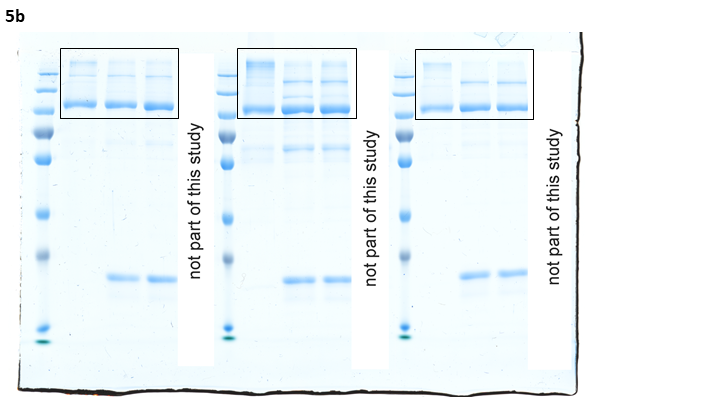

Supplement: Supplementary file 15 — Unprocessed gels. [file 41594_2023_1203_MOESM15_ESM.tif]

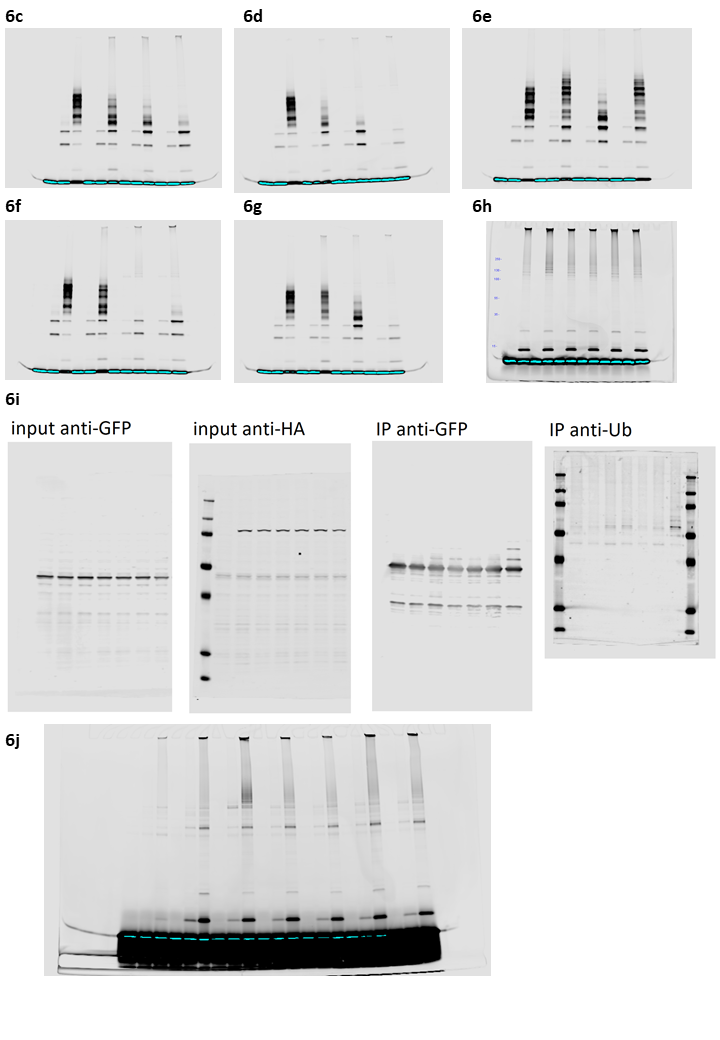

Supplement: Supplementary file 16 — Unprocessed gels and Western blots. [file 41594_2023_1203_MOESM16_ESM.tif]

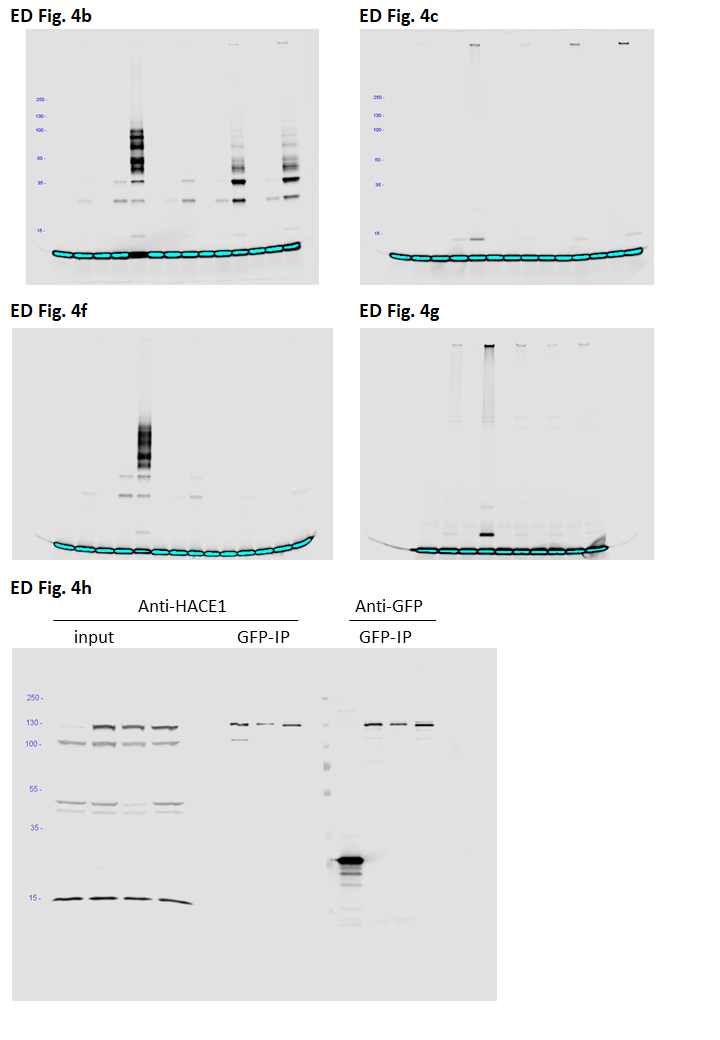

Supplement: Supplementary file 17 — Unprocessed gels and Western blots. [file 41594_2023_1203_MOESM17_ESM.tif]

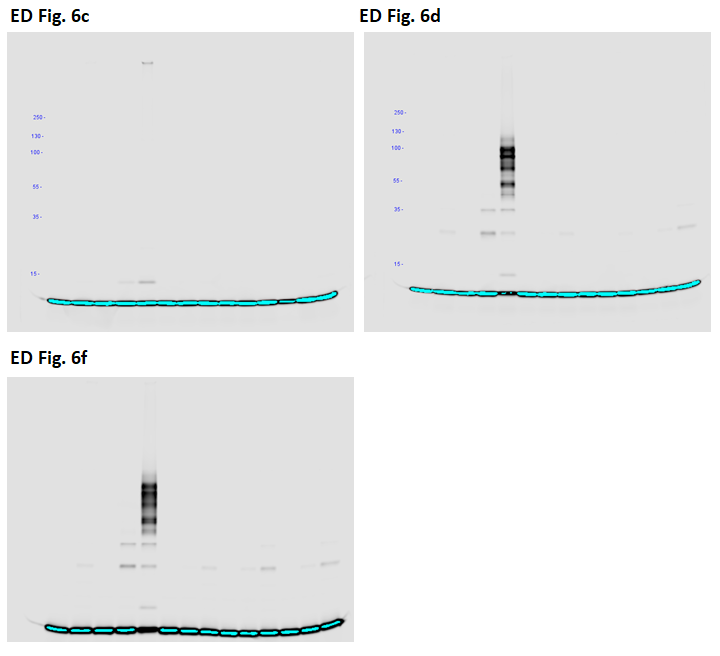

Supplement: Supplementary file 18 — Unprocessed gels. [file 41594_2023_1203_MOESM18_ESM.tif]
